# Amixicile Reduces Severity of Cryptosporidiosis but Does Not Have *In Vitro* Activity against Cryptosporidium

**DOI:** 10.1128/AAC.00718-18

**Published:** 2018-11-26

**Authors:** Luther A. Bartelt, David T. Bolick, Glynis L. Kolling, Erin Stebbins, Christopher D. Huston, Richard L. Guerrant, Paul S. Hoffman

**Affiliations:** a Division of Infectious Diseases, Department of Medicine, University of North Carolina at Chapel Hill, Chapel Hill, North Carolina, USA; b Center for Gastrointestinal Biology and Disease, Department of Medicine, University of North Carolina at Chapel Hill, Chapel Hill, North Carolina, USA; c Division of Infectious Diseases and International Health, Department of Medicine, University of Virginia, Charlottesville, Virginia, USA; d Division of Infectious Diseases, Department of Medicine, University of Vermont, Burlington, Vermont, USA

**Keywords:** Cryptosporidium, amixicile, environmental enteropathy, malnutrition, nitazoxanide

## Abstract

Cryptosporidium species cause significant morbidity in malnourished children. Nitazoxanide (NTZ) is the only approved treatment for cryptosporidiosis, but NTZ has diminished effectiveness during malnutrition. Here, we show that amixicile, a highly selective water-soluble derivative of NTZ diminishes Cryptosporidium infection severity in a malnourished mouse model despite a lack of direct anticryptosporidial activity. We suggest that amixicile, by tamping down anaerobes associated with intestinal inflammation, reverses weight loss and indirectly mitigates infection-associated pathology.

## Abstract (precis)

Cryptosporidium species cause significant morbidity in malnourished children. Nitazoxanide (NTZ) is the only approved treatment for cryptosporidiosis, but NTZ has diminished effectiveness during malnutrition.

## INTRODUCTION

Cryptosporidium species are intestinal apicomplexan protozoa that cause significant global morbidity and mortality in young children (1). Even when asymptomatic, childhood Cryptosporidium infections are associated with impaired growth attainment (2). Syndromic-based diarrhea interventions may be ineffective for cryptosporidiosis, and targeted anticryptosporidial therapeutics are unavailable, especially in resource-limited settings (2). Nitazoxanide (NTZ) is currently the only FDA-approved drug for treating cryptosporidiosis. Early clinical studies of NTZ in immunocompetent children demonstrated decreased duration of diarrhea and 75% parasitological cure (3). However, parasitological cure occurred in only 52% of malnourished children (4), and even prolonged durations of NTZ were ineffective during HIV coinfection (5). Similarly, NTZ does not consistently treat Cryptosporidium infection in several murine models, including immunodeficient knockout mice that develop relapsing disease (6) and malnourished mice that spontaneously clear infection (7, 8). Finally, despite its *in vitro* activity, NTZ only partially clears Cryptosporidium infections in piglet models (9).

**FIG 1:**
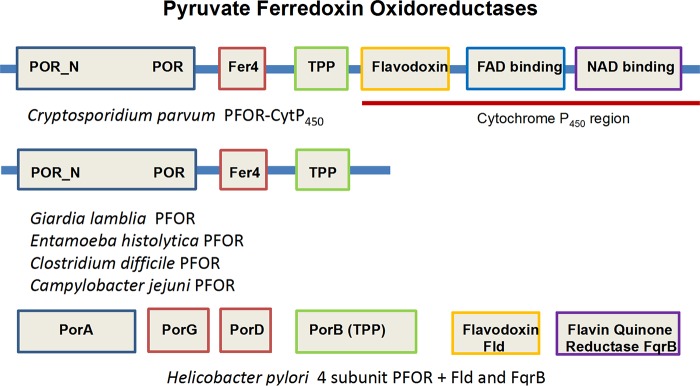
The C. parvum PFOR (GenBank accession number AF208233) is a hybrid enzyme with C-terminal cytochrome P450 that oxidizes pyruvate with reducing equivalents internally transferred through flavodoxin to NADPH oxidase that produces NADPH (11). Other PFORs transfer reducing equivalents through ferredoxin to electron acceptors to produce hydrogen. The 4-subunit H. pylori PFOR and the single-unit PFOR of C. jejuni also produce NADPH via flavodoxin (Fld) and NADPH oxidase FqrB (33) with functional evolutionary similarities with the C. parvum enzyme, including an inability to reduce the redox-active prodrug metronidazole. Not to scale.

It is unusual for an antibiotic to work in humans but not in established preclinical animal models, which has raised questions about its mechanism of action. NTZ is a broad-spectrum antiparasitic drug that inhibits pyruvate:ferredoxin oxidoreductase (PFOR) through targeting the thiamine pyrophosphate vitamin cofactor of PFOR (10). Anaerobic bacteria and parasites that rely on PFOR as an essential enzyme for central metabolism are highly susceptible to nitazoxanide. In the development of NTZ, the PFOR of Cryptosporidium was never validated by a direct enzyme assay (10). The Cryptosporidium PFOR is a hybrid enzyme containing a C-terminal cytochrome P450 protein, composed of flavodoxin and NADPH oxidase (Fig. 1) (11). This atypical arrangement appears to connect the oxidative decarboxylation of pyruvate with the reduction of NADP, thus bypassing the typical ferredoxin/hydrogenase redox route. NTZ impairs the invasion of Cryptosporidium species in epithelial cell monolayers (12); however, given the off-target effects of NTZ, such as anticancer (13), antiviral (14), and non-PFOR antiparasitic activities (15, 16), one cannot rule out the possibility that the hybrid PFOR of Cryptosporidium is not the target of NTZ. Furthermore, with the exception of the apicomplexan (Cryptosporidium) group, most pathogens that are susceptible to NTZ are also susceptible to metronidazole. Since PFOR is associated with the mode of action of both drugs, we sought to explain the basis for the exception.

Amixicile (VPC162134) is a recently developed low-toxicity water-soluble derivative of NTZ, with high systemic bioavailability (17). Amixicile was developed by replacing the 2-acetoxy group with propylamine, leading to a more water-soluble and noncytotoxic drug (18). Compared with only 30% absorption of the active NTZ metabolite tizoxanide, nearly 100% of amixicile is absorbed (17). Amixicile is a highly selective inhibitor of PFOR in anaerobes (19) and Helicobacter pylori (17, 20), and it is much more active than NTZ against other PFOR-producing pathogens, including Clostridium difficile, H. pylori, Campylobacter jejuni, and periodontal disease-promoting anaerobes (18–20). Amixicile is effective against C. difficile and H. pylori infections in mice (17), but its effect on Cryptosporidium species and malnutrition is unknown.

In order to determine the effectiveness of amixicile against Cryptosporidium species, we used previously published models of *in vivo* challenge during murine protein malnutrition (PM) (8, 21) and *in vitro* epithelial cell monolayer inhibition assays (6, 22).

All animal studies were performed at the University of Virginia with a protocol approved in accordance with the Institutional Animal Care and Use Committee (IACUC) policies of the University of Virginia (protocol number 3315). Briefly, weaned C57BL/6 mice (Jackson Laboratories) fed a 2% protein deficient diet (Research Diets) that recapitulates features of cryptosporidiosis in malnourished children, including dose-dependent disease severity (8), were challenged with 5 × 10^7^ Cryptosporidium parvum oocysts (Iowa isolate; Waterborne, Inc., New Orleans, LA). Similar to other Cryptosporidium murine models recently developed for preclinical anticryptosporidial drug testing (6), C. parvum fecal shedding was determined by quantitative PCR (qPCR; 18S rRNA target) (21) that was present only after challenge with viable, but not heat-inactivated oocysts (8). Shedding in this model is greatest during the first 3 days postchallenge, coincident with rapid weight loss. Parasites are detectable for 7- to 11-days postchallenge, but unlike immunodeficient murine knockout models, there is no relapse phase (6). Statistical analyses (two-way analysis of variance [ANOVA], including Bonferroni posttest analysis of repeated measures for growth where appropriate) were performed with GraphPad Prism 7.0 software.

**FIG 2:**
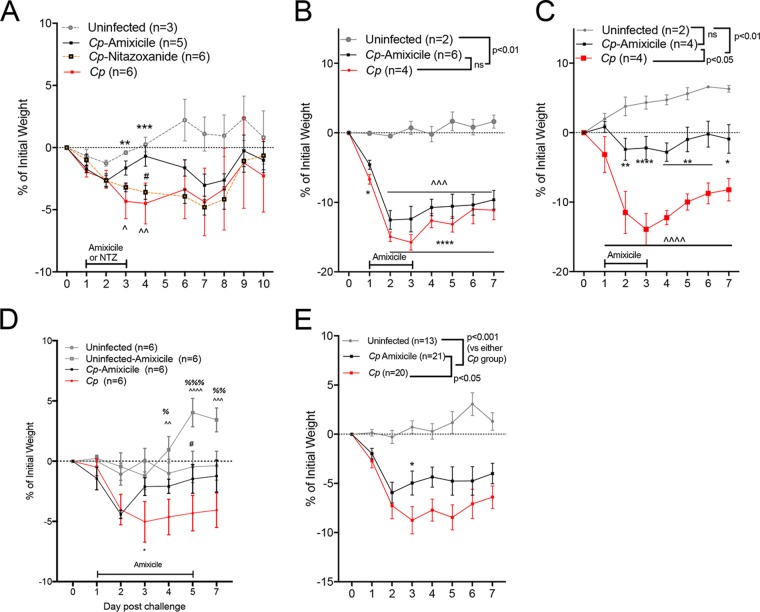
Amixicile partially reduces the severity of cryptosporidiosis during malnutrition. (A) Experiment 1. Amixicile or nitazoxanide (NTZ) was administered by orogastric gavage at equivalent doses (100 mg/kg/day) to protein-malnourished weaned mice beginning 1 day after C. parvum oocyst challenge (5 × 10^7^ oocysts in all experiments) and continued through 3-day postchallenge. Growth is shown as percent initial weight beginning on the day of C. parvum challenge (**, *P* < 0.01 and ***, *P* < 0.001 for Uninfected versus *Cp*; ^, *P* < 0.05 and ^̂, *P* < 0.01 for *Cp* versus *Cp*-Amixicile; ^#^, *P* < 0.05 for *Cp*-Amixicile versus *Cp*-NTZ). (B, C) Experiments 2 and 3. Growth is shown as percentage of weight on the day of C. parvum challenge in two separate experiments. Amixicile was administered orally at 100 mg/kg/day for 3 days beginning 1 day after challenge with C. parvum oocysts. (B) *, *P* < 0.05 and ****, *P* < 0.0001 for *Cp* versus Uninfected; ^̂̂, *P <* 0.001 for *Cp*-Amixicile versus Uninfected. (C) *, *P* < 0.05; **, *P* < 0.01; ***, *P* < 0.001; ****, *P* < 0.0001 for *Cp*-Amixicile versus *Cp*. ^̂̂̂, *P* < 0.0001 for *Cp* versus Uninfected. (D) Experiment 4. Growth in weaned mice fed a protein deficient diet as percent initial change beginning on the day of C. parvum challenge. Amixicile was administered orally at 100 mg/kg/day on day 1 to 5 postchallenge. *, *P* < 0.05 for *Cp* versus Uninfected; ^̂, *P* < 0.01; ^̂̂, *P* < 0.01; and ^̂̂̂, *P* < 0.001 for Uninfected-Amixicile versus *Cp*-Amixicile. ^%^, *P* < 0.05; ^%%^, *P* < 0.01; and ^%%%^, *P* < 0.001 for Uninfected-Amixicile versus *Cp*. ^#^, *P* < 0.05 for Uninfected-Amixicile versus Uninfected. (E) Growth curves for Uninfected, *Cp*, and *Cp*-Amixicile groups combined across all four experiments (*, *P* < 0.05 for *Cp* versus *Cp*-Amixicile; 3 dpi). For growth curves B, C, and E, brackets designate comparisons of growth curves throughout the time course (two-way ANOVA).

Amixicile (diluted in deionized water) or NTZ (as a pediatric solution [Alinia; Romark Pharmaceuticals, Tampa, FL] diluted in deionized water) was given at equivalent doses of 100 mg/kg/day of body weight (7, 18) by orogastric gavage once daily beginning 1-day postinfection (1 dpi) and continued through 3 dpi. We performed four independent experiments investigating the effectiveness of amixicile against C. parvum challenge *in vivo*. First, in direct comparison, amixicile, but not NTZ, partially rescued mice from early weight loss (0.69% versus 4.5% weight loss at 4 dpi; *P* < 0.05) (Fig. 2A). C. parvum stool shedding on 1 (collected prior to treatment), 5, or 7 dpi was similar in all challenged mice regardless of treatment (see Fig. S1A in the supplemental material). In one of two separate follow-up experiments (Fig. 2B and C), amixicile partially attenuated severity of weight loss (Fig. 2C). C. parvum shedding was not conclusively reduced in amixicile-treated animals (see Fig. S1B and C in the supplemental material).

Since neither PFOR inhibitor eliminated C. parvum, we hypothesized that the primary effect of amixicile was to benefit the malnourished host. Whereas uninfected mice treated for 3 days with NTZ exhibited weight loss, mice treated with amixicile did not (see Fig. S2 in the supplemental material). In a fourth experiment, extending amixicile treatment to 5 days promoted weight gain by malnourished uninfected animals (Fig. 2D). Composite data for untreated and amixicile-treated infected groups compared with untreated uninfected controls for all *in vivo* experiments demonstrated a modest growth benefit in amixicile-treated animals (*P* < 0.05 for untreated C. parvum-challenged mice) (Fig. 2E) that was most apparent as a rescue from weight loss upon initiation of amixicile (see Fig. S3 in the supplemental material).

**FIG 3:**
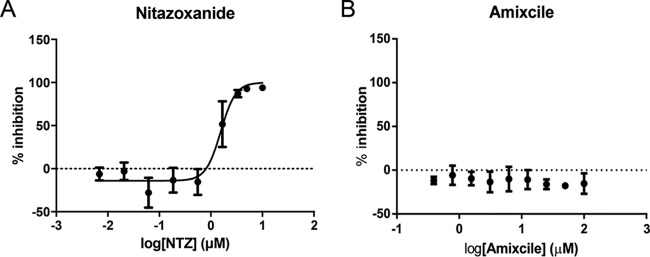
Amixicile, a selective PFOR inhibitor, has no *in vitro* activity against Cryptosporidium parasites. Nitazoxanide or amixicile was administered at concentrations ranging from log −2.16 to 1 µM or log −0.216 to 2.0 µM, respectively. Inhibition was determined in a cell-based high-throughput screening assay using confluent HCT-8 monolayers inoculated with 5.5 × 10^3^ C. parvum oocysts. (A) Nitazoxanide inhibition curve EC_50_, 1.55 µM (range, 1.31 to 1.84 µM); EC_90_, 3.23 µM (range, 2.32 to 4.50 µM). (B) Amixicile inhibition curve. Each graph shows representative results from one of two experiments, with multiple technical replicates for each data point (NTZ, *n* = 5; Amixicile, *n* = 4).

For *in vitro* testing, we compared both PFOR inhibitors using an assay which involves inoculating HCT-8 cells (ATCC) grown to confluence with 5.5 × 10^3^ excystation-primed C. parvum oocysts (Bunchgrass Farms, Deary, ID) (12), staining for epifluorescence microscopy after a 48-h incubation, and counting parasites and host cells using automated microscopy (12). The 50% effective concentration (EC_50_) of NTZ was 1.55 (range, 1.31 to 1.84) µM, but across repeated experiments, amixicile had no anticryptosporidial activity, even at ≥100 µM (Fig. 3A and B). Since NTZ and amixicile are identical in docking simulations with PFOR and amixicile is highly selective for PFOR, it is likely that the PFOR-CytP450 is not a target for either drug (20). Our suggestion that PFOR/CytP450 is an unlikely target of NTZ is consistent with a growing view that other inhibitory mechanisms are involved (16). Although off-target activities attributed to NTZ against Cryptosporidium species are not known, amixicile is a more selective PFOR inhibitor which might explain why nitazoxanide, but not amixicile, is active against C. parvum *in vitro*. Off-target promiscuous activities of NTZ against Cryptosporidium species may contribute to the variable outcomes observed clinically with this drug.

Promising new anticryptosporidial therapeutics rapidly eliminate parasites in other animal models (6, 23, 24). In contrast, amixicile mitigated weight loss, a very important disease feature of cryptosporidiosis, but did so without significantly accelerating parasite elimination, which suggests that its effect on malnutrition in cryptosporidiosis is indirect. Cryptosporidium infections (25) and other intestinal microbial disruptions (26) are increasingly linked to malnutrition in both children (27) and murine models (28–30). Together, these microbial exposures may result in a multimicrobial condition of chronic intestinal inflammation and injury termed environmental enteric dysfunction (EED) (25, 26, 31). In murine models of EED, there is an overabundance of anaerobic Bacteroidales members in the upper small intestine during malnutrition, and inclusion of Bacteroides spp. with a cocktail of Escherichia coli isolates was necessary to enhance intestinal inflammation (26).

Given the spectrum of amixicile activity (17), its absence in stools, and its apparent ability to concentrate in regions of local intestinal inflammation due to serum leakage (17, 18), we suggest amixicile likely targets the offending mucosal-associated anaerobes, and, thus, reduces inflammation and improves barrier function and absorption that collectively leads to weight gain and elimination of the parasite. NTZ, which concentrates in the gut lumen, does not reverse weight loss in this model. Supporting this multimicrobial pathogenesis hypothesis, Cryptosporidium infection as well as other enteric infections in this model of PM can exacerbate diet-dependent disruptions in gut-bacterium-derived metabolites, such as trimethylamine (TMA) and trimethylamine oxide (TMAO) (28, 30), that are also altered in some malnourished children (29, 32). These observations are consistent with previous findings that amixicile, which does not alter the gut microbiome, eliminates C. difficile and H. pylori infections in murine models (17, 18).

In conclusion, the NTZ derivative amixicile, a highly selective water-soluble PFOR inhibitor that appears to concentrate in sites of local inflammation (18), reversed weight loss in the malnourished host, despite no evidence of *in vitro* anticryptosporidial activity. Furthermore, neither NTZ nor amixicile resulted in a definitive reduction of parasite shedding *in vivo*. Unlike currently available antibiotic regimens for treating malnutrition/EED (31) that could cause collateral loss of potentially beneficial microbiota, amixicile spares the intestinal microbiota of mice (17), and may, therefore, preserve healthy luminal microbial density while selectively targeting subpopulations of anaerobes causing active infection. Thus, demonstrating that Cryptosporidium parasites are resilient to PFOR inhibition *in vitro*, but that infection can be attenuated by the PFOR inhibitor amixicile, led us to propose a model of mixed-microbial pathogenesis during cryptosporidiosis and malnutrition (30) and the potential role for both direct and indirect therapies to address the management of this emerging global pathogen.

## Supplementary Material

Supplemental file 1
