# Supplementary material for: Amixicile Reduces Severity of Cryptosporidiosis but Does Not Have In Vitro Activity against Cryptosporidium
Source: Antimicrob Agents Chemother. 2018 Nov 26;62(12):e00718-18. doi: 10.1128/AAC.00718-18 (PMC6256802; doi:10.1128/AAC.00718-18)
Supplement: Supplemental file 1 [file zac012187677s1.pdf]

Supplemental Figure 1

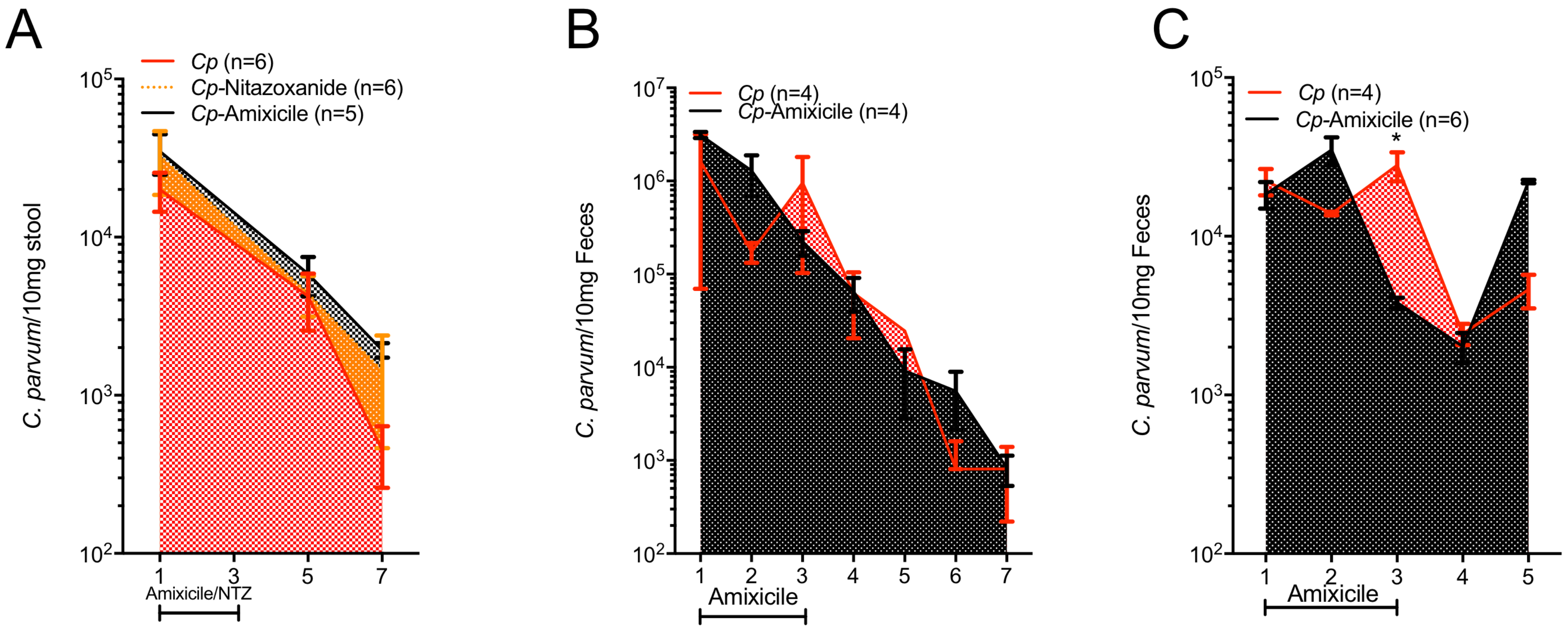

Supplemental Figure 2

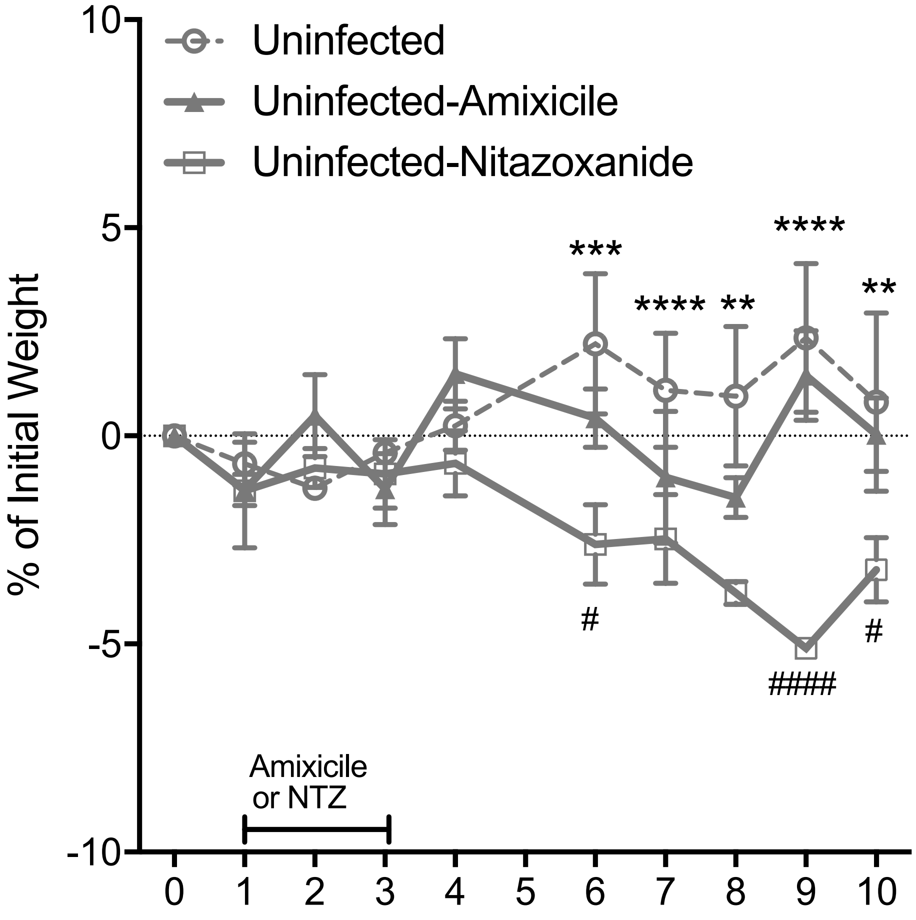

Supplemental Figure 3

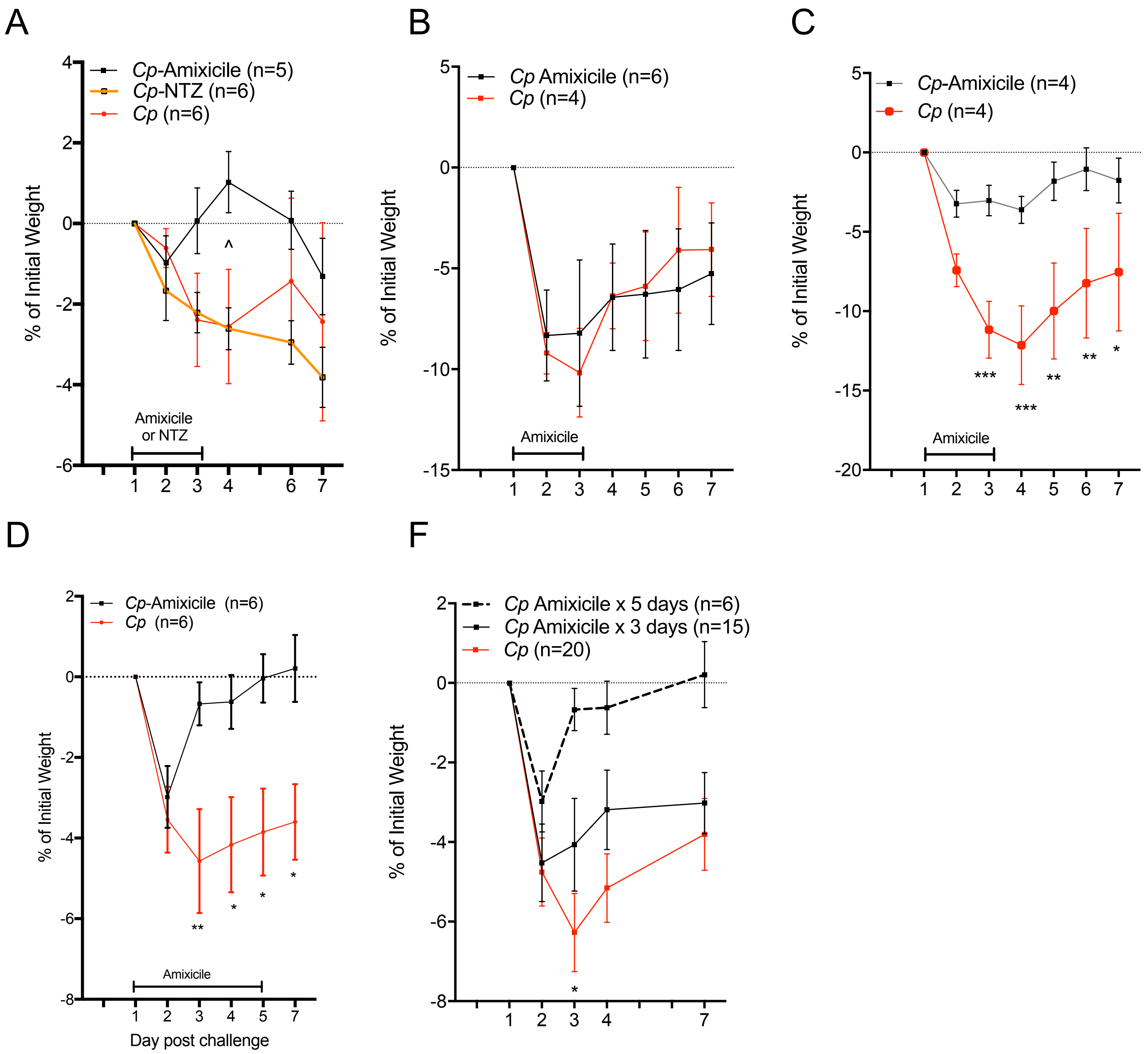

Supplemental Figures Legends

Supplemental Figure 1. *C. parvum* stool shedding (qPCR of the 18S rRNA target) in untreated (*Cp*), Nitazoxanide-treated (*Cp*-Nitazoxanide), and Amixicile-treated (*Cp*-Amixicile) groups beginning one day after challenge with 5 x 10^7 oocysts in three separate experiments (n=4-6 mice per group as indicated). A) *C. parvum* stool shedding by qPCR on days 1, 5, and 7 post-challenge. B, C) Daily *C. parvum* stool shedding for the first 7 (B) and 5 (C) days post-challenge. *P<0.05, day 3 *Cp* vs. *Cp*-Amixicile (C).

Supplemental Figure 2. Amixicile is better tolerated than nitazoxanide during protein malnutrition. A) Growth as % initial change in uninfected mice beginning one day prior to treatment with either Amixicile or Nitazoxanide at 100 mg/kg/mouse as indicated. (**P<0.01, **P<0.001, and **** P<0.0001 for uninfected vs. uninfected-NTZ; and $^{\#}$P<0.05 and $^{\#\#\#\#}$P<0.001 for uninfected-amixicile vs. uninfected-NTZ).

Supplemental Figure 3. Initiation of amixicile corresponds with accelerated growth recovery after *C. parvum* challenge. A-D) Growth as % weight change starting on the day of NTZ or amixicile treatment (day 1 post-*C. parvum* challenge) and through 7 days post-challenge in each of the 4 individual experiments depicted in Figure 2. Groups shown are untreated *C. parvum* challenged (*Cp*) compared with groups treated with amixicile (*Cp*-Amix) and in A), NTZ (*Cp*-NTZ) (n=4-6 mice/group as indicated). For each experiment, *P<0.05, **P<0.01, ***P<0.001 for *Cp* vs *Cp*-Amix. ^P<0.05 for *Cp*-Amix vs. *Cp*-NTZ. E) Composite growth as % initial change beginning on the day of treatment with amixicile for either three days (figures A-C) or 5 days (figure D) as indicated. *P<0.05 for *Cp* vs *Cp*-Amixicile x 5 days.
